# Predominant gut *Lactobacillus murinus* strain mediates anti-inflammaging effects in calorie-restricted mice

**DOI:** 10.1186/s40168-018-0440-5

**Published:** 2018-03-21

**Authors:** Fengwei Pan, Liying Zhang, Min Li, Yingxin Hu, Benhua Zeng, Huijuan Yuan, Liping Zhao, Chenhong Zhang

**Affiliations:** 1 State Key Laboratory of Microbial Metabolism, School of Life Sciences and Biotechnology, Shanghai Jiao Tong University, Shanghai, 200240 China; 2 Department of Laboratory Animal Science, College of Basic Medical Sciences, Third Military Medical University, Chongqing, 400038 China; 3 Henan Provincial People’s Hospital, Zhengzhou, 450003 Henan Province China

**Keywords:** Calorie restriction, Gut microbiota, Chronic inflammation, Lifespan, *Lactobacillus murinus*

## Abstract

**Background:**

Calorie restriction (CR), which has a potent anti-inflammaging effect, has been demonstrated to induce dramatic changes in the gut microbiota. Whether the modulated gut microbiota contributes to the attenuation of inflammation during CR is unknown, as are the members of the microbial community that may be key mediators of this process.

**Results:**

Here, we report that a unique *Lactobacillus*-predominated microbial community was rapidly attained in mice within 2 weeks of CR, which decreased the levels of circulating microbial antigens and systemic inflammatory markers such as tumour necrosis factor alpha (TNF-α). *Lactobacillus murinus* CR147, an isolate in the most abundant operational taxonomic unit (OTU) enriched by CR, downregulated interleukin-8 production in TNF-α-stimulated Caco-2 cells and significantly increased the lifespan and the brood size of the nematode *Caenorhabditis elegans*. In gnotobiotic mice colonized with the gut microbiota from old mice, this strain decreased their intestinal permeability and serum endotoxin load, consequently attenuating the inflammation induced by the old microbiota.

**Conclusions:**

Our study demonstrated that a strain of *Lactobacillus murinus* was promoted in CR mice and causatively contributed to the attenuation of ageing-associated inflammation.

**Electronic supplementary material:**

The online version of this article (10.1186/s40168-018-0440-5) contains supplementary material, which is available to authorized users.

## Background

Calorie restriction (CR) without malnutrition, which can extend lifespan and retard age-related diseases in many different organisms [1], has been demonstrated to modulate the gut microbiota and the profiles of microbial metabolites in serum or urine [2–7]. In a life-long mouse trial, 30% CR established a unique gut microbiota structure dominated by *Lactobacillus* spp. and reduced the bacterial antigen load in the serum of middle-aged mice [2]. In humans, 10 weeks of a CR diet plus physical activity shifted the composition of the gut microbiota in overweight adolescents [3]. A longer CR intervention (which was mainly due to a significant reduction in total carbohydrates and fat content) that lasted 1 year led to an increase in fecal Bacteroidetes and a decrease in Actinobacteria [4]. Although not fully elucidated, these particular changes in the gut microbiota may plausibly impact or mediate the beneficial effects associated with CR.

A low-grade, systemic and chronic inflammatory condition has been recognized as a crucial pathological process underlying metabolic syndrome and accelerated ageing (inflammaging) [8, 9]. Compelling evidence suggests that CR may exert its beneficial actions through the attenuation of the inflammatory state associated with ageing and age-related diseases [10–12]. The gut microbiota, which can be directly modulated by CR, has been demonstrated to play a critical role in the pathogenesis and development of systemic inflammation. High-fat diet-induced dysbiosis of the gut microbiota damaged the gut barrier and resulted in higher levels of lipopolysaccharide (LPS) in the host blood, causing inflammation and, consequently, obesity and insulin resistance [13–15]. A recent study showed that intestinal permeability and the levels of circulating bacterial products increased with age in mice due to age-associated microbial dysbiosis, which promoted the circulating pro-inflammatory cytokine levels [16]. Whether the modulated gut microbiota contributes to the attenuation of inflammation by CR became an interesting question, as did which members of the microbial community are the key mediators.

Here, we report a unique *Lactobacillus murinus*-dominated microbial community that was rapidly attained in mice within 2 weeks of CR, which decreased the levels of circulating microbial antigens and marker of systemic inflammation. A specific strain of *Lactobacillus murinus* in the most abundant operational taxonomic unit (OTU) was isolated from the feces of CR mice and shown to be a key mediator of the anti-ageing and anti-inflammatory effects of CR in various experimental systems, including Caco-2 cells, *Caenorhabditis elegans* and old microbiota-colonized gnotobiotic mice.

## Results

### Short-term calorie restriction improves metabolic health and alters the gut microbiota in mice

**Fig. 1:**
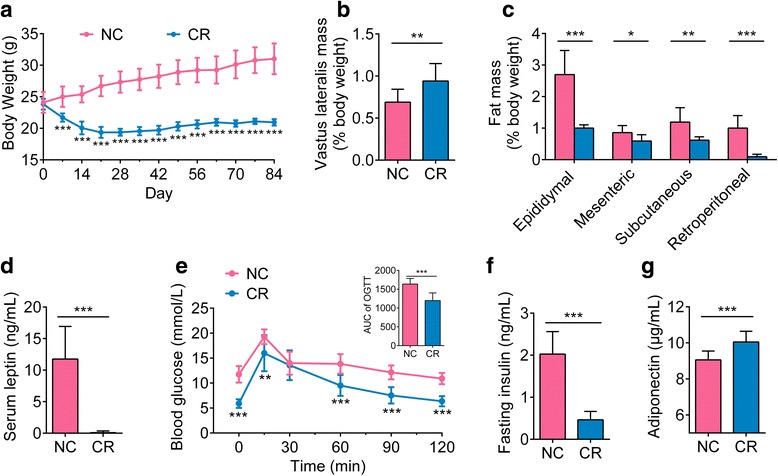
Short-term calorie restriction improves body composition and metabolic health in mice. **a** Body weight curves. **b** Vastus lateral mass (% body weight). **c** Fat mass (% body weight). **d** Serum leptin. **e** Curves of oral glucose tolerance test (OGTT) and areas under the curve (AUC). **f** Fasting serum insulin. **g** Serum adiponectin. Data are presented as the mean ± standard deviation. Unpaired *t* test (two-tailed) was used to analyse variation between the NC and CR groups at the same time point. *P < 0.05, **P < 0.01 and ***P < 0.001 vs the NC group at the same time point. *n* = 10 for both groups for all analyses except *n* = 9 in the NC group for **b**

To observe the effect of short-term CR on mice, we randomly assigned mice into two groups: the first group received a normal chow diet ad libitum (NC group), while the second was fed 70% of the ad libitum chow (CR group) for 12 weeks. Compared with the NC group, the CR mice exhibited a significant decrease in body weight after just 1 week (Fig. 1a). After 84 days, CR animals had a higher vastus lateralis mass (Fig. 1b) and a lower fat mass (Fig. 1c) as a percentage of body weight. Lower serum levels of leptin, a fat-derived hormone, also indicated less fat accumulation in the CR mice (Fig. 1d). CR significantly improved glucose-insulin homeostasis in mice, with reduced fasting blood glucose levels, enhanced glucose clearance in the oral glucose tolerance test (OGTT) (Fig. 1e) and lowered fasting serum insulin (Fig. 1f). Additionally, the levels of adiponectin, an anti-inflammatory adipokine, were significantly higher in the CR group than in the NC group (Fig. 1g). All these results revealed that short-term CR improved body composition and metabolic health, which was consistent with the improved midlife metabolic phenotypes observed during life-long CR intervention [17].

**Fig. 2:**
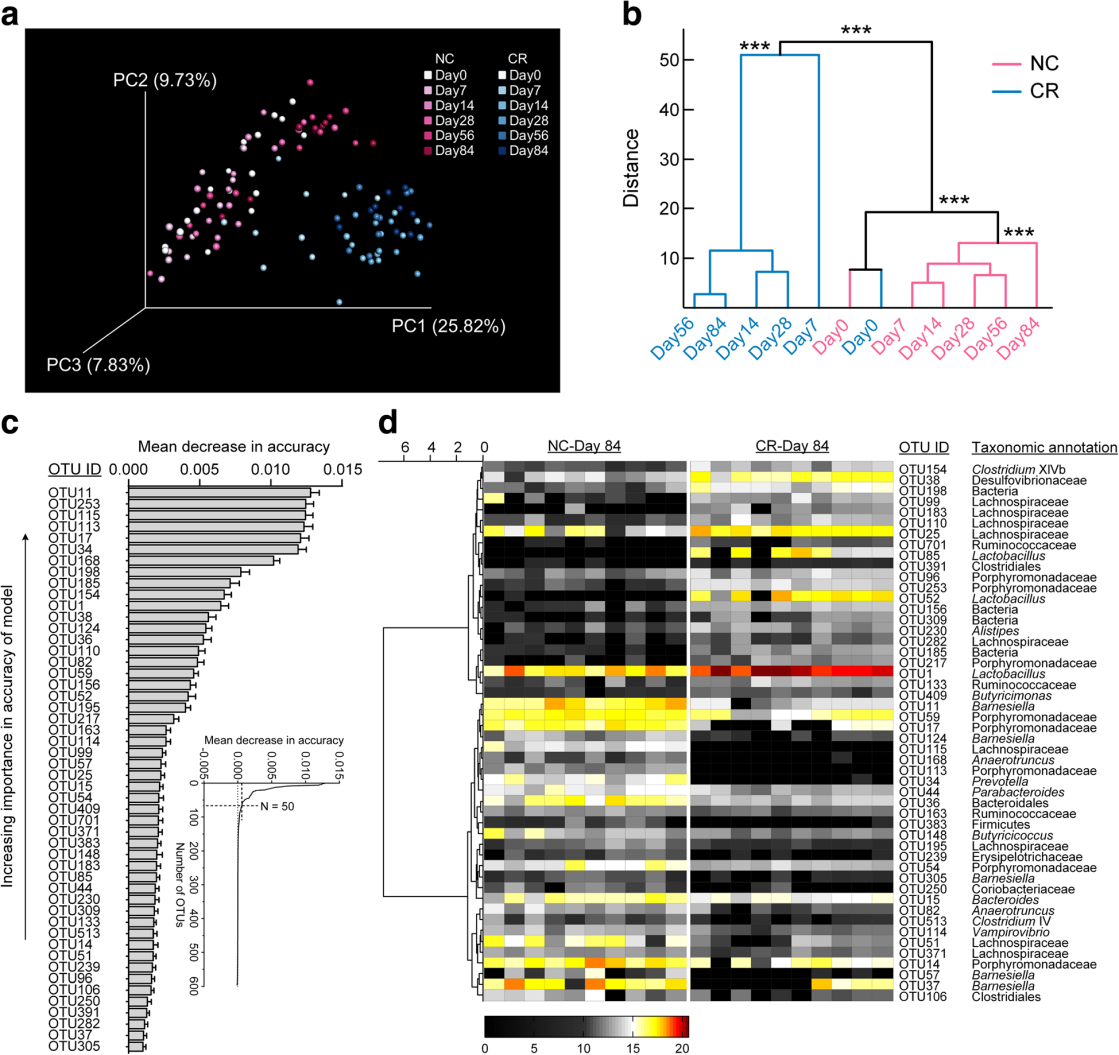
Short-term calorie restriction alters the gut microbiota in mice. **a** PCoA based on the Bray-Curtis distance. **b** Clustering of the gut microbiota between different groups calculated with multivariate analysis of variances (MANOVA) test using the first 25 principal components (PCs) (accounting for 80.73% of total variations) of PCoA based on Bray-Curtis distance. ***P < 0.001. **c** The rank of the 50 OTU-level phylotypes identified as key variables for differentiation between the gut microbiota of the CR group and that of the NC group by random forests. Day 84 samples from the NC and CR groups were included for discrimination. OTUs were ranked in descending order of important scores, which were determined by estimating the mean decrease in the accuracy (MDIA) when a specific OTU was removed from the set of predictors. Data are shown as the mean ± s.e.m. **d** Heat map of the 50 key OTUs. The colour of the spots in the panel represents the relative abundance (normalized and log2-transformed) of the OTU in each sample. The OTUs were organized by Spearman’s correlation analysis based on their relative abundances. The genus-level taxonomic classifications of the OTUs are shown on the right. *n* = 10 for both groups at all time points except *n* = 9 for the CR group at day 7 and day 28 and *n* = 8 for the NC group at day 28

To determine how the gut microbiota was modulated by short-term CR, the overall structural changes of the gut microbiota were profiled (Additional file 1). The number of observed OTUs and Shannon diversity index revealed a decrease in the richness and diversity of the gut microbiota in the first month of CR (Additional file 2). As revealed by principal coordinate analysis (PCoA) of the Bray-Curtis distances based on the OTU data, the overall structure of the gut microbiota was significantly shifted after just 7 days of CR (Fig. 2a; *P* < 0.001 with PerMANOVA test, 9999 permutations), and the gut microbiota continued to change from day 7 to day 14; however, no significant differences occurred from day 14 through day 84 in the CR mice (Fig. 2b), indicating that the gut microbiota became dynamically stable after 14 days of CR.

Random forests were employed to construct a classification model between the NC and CR groups at day 84 (the classification error with leave-one-out cross validation was zero), and 50 OTUs were highly predictive for the differentiation between the gut microbiota of the CR group and that of the NC group (Fig. 2c). Overall, 28 of the 50 OTUs decreased in the CR mice, whereas the remaining 22 OTUs increased (Fig. 2d and Additional file 3). Some of these phylotypes in the same family or genus showed different behaviours. For example, among the 15 key OTUs in the family Porphyromonadaceae, 4 OTUs were significantly enriched while 11 OTUs were decreased by CR. Moreover, if the identified OTUs in the same family or genus were all increased or decreased by CR, they showed different dynamic changes during short-term CR. Notably, three OTUs (OTU1, OTU52 and OTU85) in the genus *Lactobacillus* were all significantly enriched by CR, but OTU1 was promoted the most and became the most predominant phylotype in the bacterial communities of CR mice after 84 days (CR versus NC, 12.11 versus 2.53%; *P* < 0.001, Mann-Whitney *U* test). In our previous life-long CR study, *Lactobacillus* showed a strong positive correlation with lifespan, and the most abundant OTU in the genus *Lactobacillus* was approximately 12% of the total population in the CR mice at 62 weeks of age (versus 0.05% in ad libitum group) [2]. These findings suggest that bacteria belonging to *Lactobacillus* become prevalent phylotypes in CR mice in early life and remain predominant for a long time.

### Calorie restriction rapidly creates a unique gut microbial community predominated by *Lactobacillus* spp. in mice

**Fig. 3:**
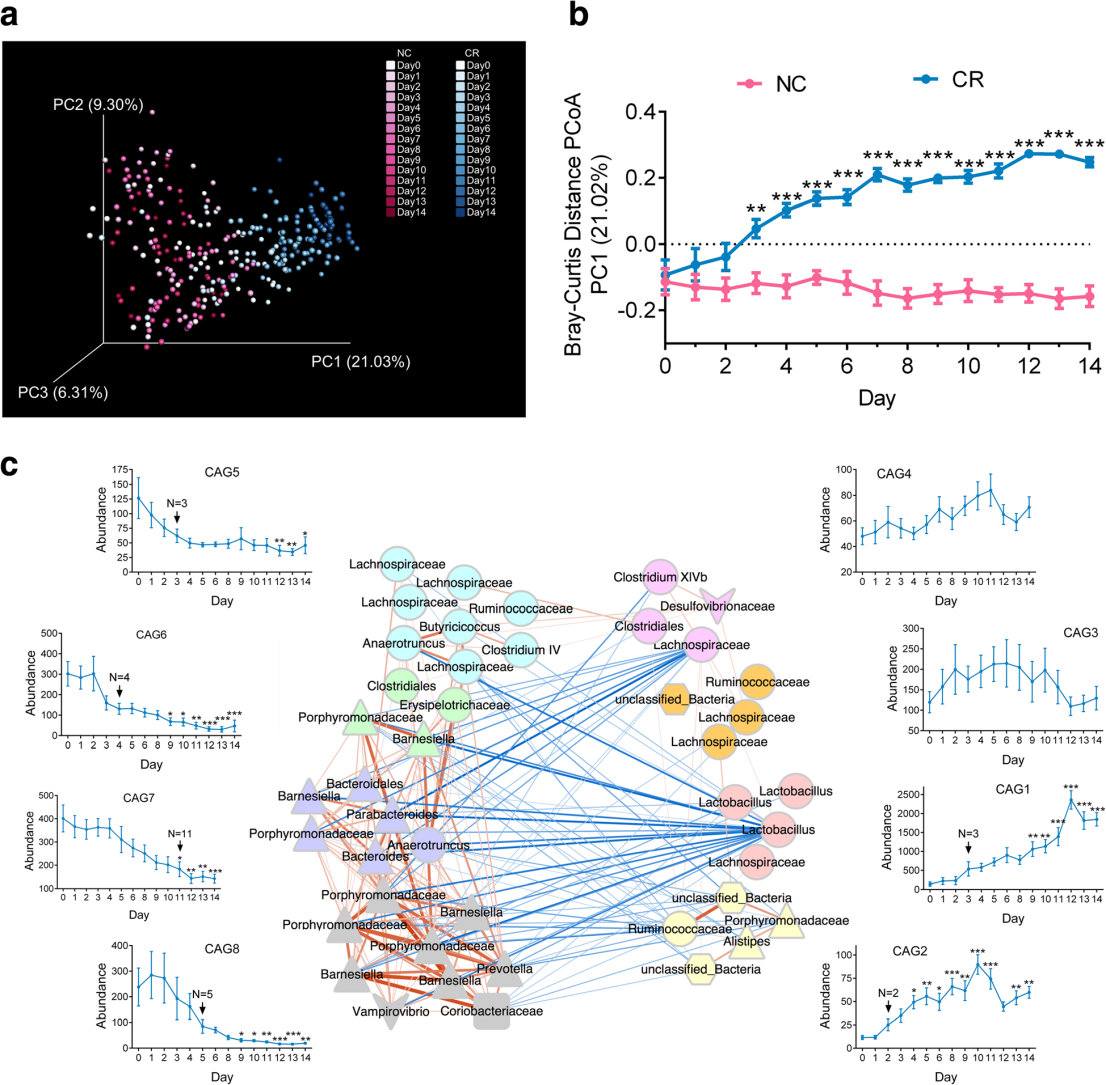
Calorie restriction rapidly creates a *Lactobacillus*-predominated gut microbial community in mice in 14 days. **a** PCoA based on Bray-Curtis distance. **b** Alteration of the gut microbiota structure along the first principal component (PC1) of the PCoA based on Bray-Curtis distance. Data were plotted as the mean ± s.e.m. Mann-Whitney *U* test (two-tailed) was used to analyse variation between the NC and CR groups at the same time point. **P < 0.01 and ***P < 0.001 vs the NC group at the same time point. **c** Co-abundance network illustrating the interactions among the 50 key OTUs in the CR group. The 50 OTUs were grouped into eight co-abundance groups (CAGs) by permutational multivariate analysis of variance (PerMANOVA) test when *P* < 0.001. Different colours and shapes of nodes represent different CAGs and phyla, respectively. The line connecting two nodes represents the correlation between the connected nodes, with the colour saturation and line width indicating the correlation magnitude: red represents a positive correlation, and blue represents a negative correlation. Only lines corresponding to correlations with a magnitude greater than 0.5 were drawn. The plots show the relative abundance of each CAG in the CR group in the first 14 days on a daily basis. Data in the plots represent the total relative abundance of all OTUs in each CAG from each sample of the CR group and are reported as the mean ± s.e.m. *N* in the plots indicates the days after calorie restriction when a specific CAG began to change, as determined by an absolute value of its logarithmic (base 2)-fold change in relative abundance greater than 1 (|log_2_-fold change| > 1). Kruskal-Wallis test followed by Dunn’s multiple comparison test was used to analyse variation relative to day 0. *P < 0.05, **P < 0.01 and ***P < 0.001 vs day 0. *n* = 10 for both NC and CR groups at all time points except *n* = 9 for the NC group at day 10 and day 11 and *n* = 9 for the CR group at day 2

The above results showed a unique gut microbiota structure in the CR mice that was created in 14 days. To reveal the rapid dynamic response of the gut microbiota to CR, we further monitored the fecal microbiota of CR mice in the first 2 weeks on a daily basis. The richness and community diversity of the gut microbiota decreased significantly after 7 days of CR (Additional file 4). The PCoA plot based on Bray-Curtis distance showed that the gut microbiota of the CR group significantly diverged from that of the NC group mainly along the first principal component (PC1), reflecting that CR was the predominant factor shifting the gut microbiota (Fig. 3a). Along PC1, a significant shift of the gut microbiota was observed after just 3 days of CR (Fig. 3b). The overall gut microbiota structure of the CR mice was significantly different from that of the NC group after day 2 (*P* < 0.05 with PerMANOVA test, 9999 permutations; Additional file 5).

We then constructed a co-abundance network to illustrate the potential interactions among the 50 OTUs that were significantly different between the CR and NC mice based on the CR group OTU data from the first two weeks. These OTUs were clustered into eight co-abundance groups (CAGs) (Fig. 3c). Here, a specific CAG was considered to be altered when its absolute value of logarithmic (base 2) fold change (|log_2_-fold change|) in relative abundance was greater than 1. Among the eight CAGs, CAG3 and CAG4 showed no changes during the first 14 days, while CAG1 and CAG2 were enriched and CAG5, 6, 7 and 8 were decreased by CR (Fig. 3c). Overall, CAG1 and CAG2 responded more rapidly to CR than the other six groups. CAG2 responded most rapidly to CR, and it began to increase from day 2 and was significantly enriched from day 4. CAG1, including three OTUs (OTU1, OTU52 and OTU85) in the genus *Lactobacillus*, was the most promoted group, which began to increase from day 3 and was significantly enriched from day 9. Moreover, OTU1 showed a strong negative correlation with the four decreased CAGs in the CR mice (Fig. 3c) and was the most predominant phylotype in the CR mice from day 3 (its relative abundance ranged from 6.51 to 28.27%) (Additional file 6).

**Fig. 4:**
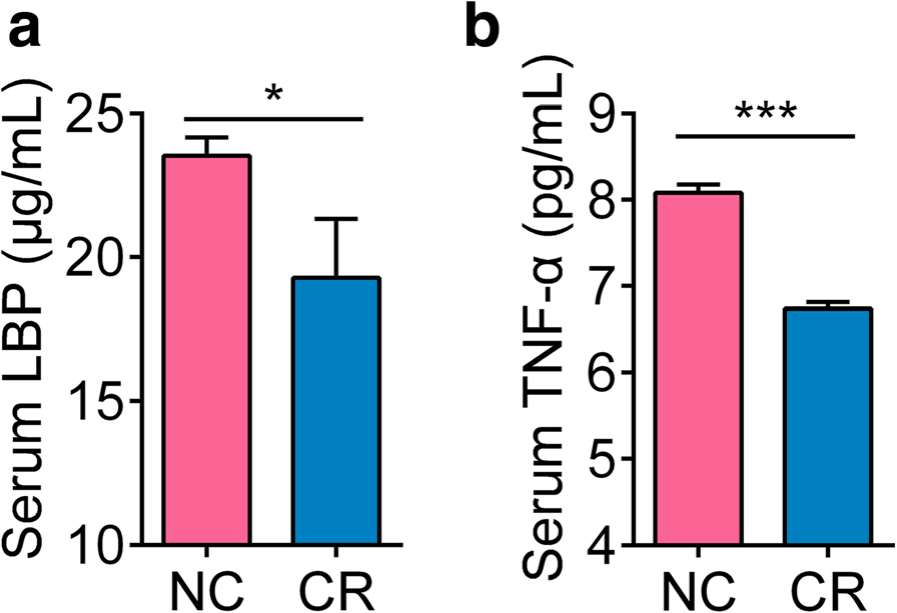
Fourteen days of calorie restriction decreased the bacterial antigen load and systemic inflammatory marker in mice. **a** Serum LBP. *n* = 10 for the NC group and *n* = 6 for the CR group. **b** Serum TNF-α. *n* = 10 for both groups. Data are shown as the mean ± s.e.m. Unpaired *t* test (two-tailed) was used to analyse variation between the NC and CR groups. *P < 0.05 and ***P < 0.001

In the recent study, Thevaranjan et al. used TNF KO mice to show that TNF-α play the critical role in gut microbiota-induced aging-associated systemic inflammation [16]. On the other hand, lipopolysaccharide (LPS)-binding protein (LBP) can bind to antigens produced by bacteria and, thus, may represent a biomarker that links bacterial antigen load in the blood and the host inflammatory response [18]. To determine whether 2 weeks of CR also decreased the bacterial antigen load and subsequent systemic inflammation, we measured the serum levels of LBP and pro-inflammatory cytokine TNF-α from animals on day 14. LBP and TNF-α levels were both significantly lower in the CR mice than in the NC mice (Fig. 4).

In summary, CR rapidly shifted the gut microbiota, creating a unique *Lactobacillus*-predominated gut microbiota, and effectively decreased the bacterial antigen load and systemic inflammatory marker in only 14 days.

### Sequence-guided isolation leads to the identification and genomic characterization of two *Lactobacillus murinus* strains enriched by CR

To investigate whether these *Lactobacillu*s spp. contribute to host health benefits induced by CR, we isolated the predominant *Lactobacillus* bacteria from the feces of CR mice. With a ‘sequence-guided isolation’ scheme, we obtained two isolates (named CR141 and CR147) that represented the most abundant OTU (OTU1) in the genus *Lactobacillu*s spp. in the CR mice (Additional file 7a, b). The similarity of the representative sequence of OTU1 and the 16S rRNA gene sequence of CR141 or CR147 was 100%. The cells of CR141 and CR147 were both rod-shaped with no flagella or other appendages under electron microscopy: the CR141 cells were about (2.0–2.1) × 0.6 μm; the CR147 cells were about (1.4–2.0) × (0.6~ 0.8) μm (Additional file 7c, d). Under anaerobic conditions, CR141 and CR147 grew to stationary phase after about 12 and 16 h, respectively, but they both decreased the pH value of the MRS medium by 1.8 when grew to stationary phase (Additional file 7e, f).

**Fig. 5:**
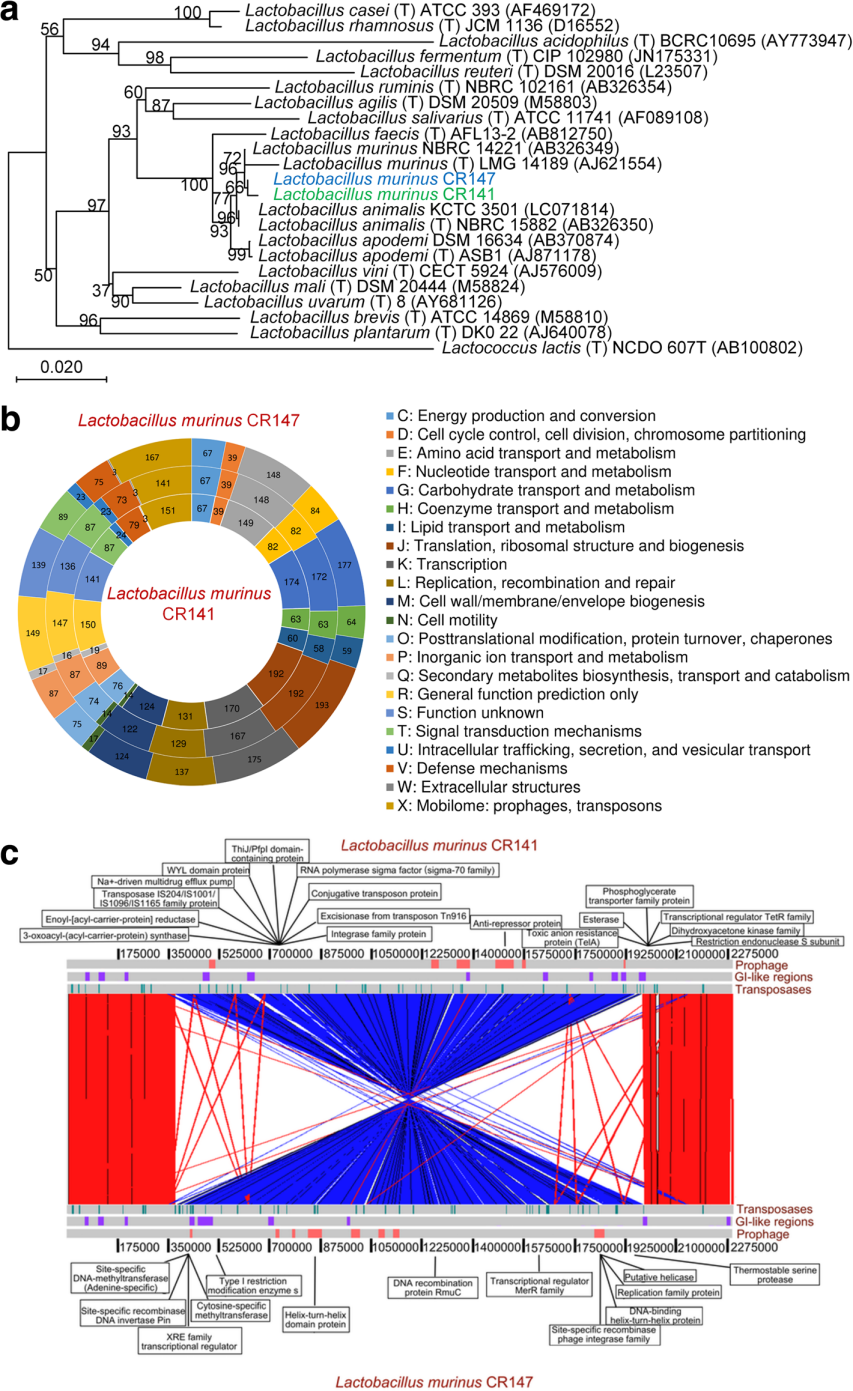
Identification and genomic characterization of the two strains of *L. murinus* enriched by calorie restriction. **a** Phylogenetic relationships of the *Lactobacillus* isolates with their relatives based on 16S rRNA gene sequences. The tree was constructed using the Neighbour-Joining method in MEGA7, and *Lactococcus lactis* NCDO 607T was used as an outgroup. The bar indicates sequence divergence. **b** Functional categories in the clusters of orthologous groups (COG) analysis of *L. murinus* CR141 and CR147. The middle circle represents the number of COG families shared by the two strains in each category. **c** Linear genomic comparison of the chromosomes of *L. murinus* CR141 and CR147. Both sequences are read left to right from the predicted replication origin. Homologous regions within the two genomes identified by BLASTN are indicated by red (same orientation) and blue (reverse orientation) bars. Prophage-related regions (red boxes), GI (genomic island)-like regions (purple boxes) and transposases (green boxes) identified by VRprofile are indicated. The strain-specific CDSs with assigned functions are also labelled

Strains CR141 and CR147 were both identified as members of the genus *Lactobacillus*, forming a subcluster in the *L. salivarius* phylogenetic group, and were highly related to *L. murinus*, *L. animalis*, *L. apodemi* and *L. faecis*, as determined by phylogenetic analysis using 16S rRNA gene sequences (> 97% sequence similarity in 16S rRNA gene) (Fig. 5a and Additional file 8). The complete genome sequences of CR141 and CR147 were then compared with publicly available genomes of their phylogenetic relatives by means of average nucleotide identity (ANI) calculations. Using ANI, the border for species delimitation can be set at 95–96% [19]. Strains CR141 and CR147 shared ANI values with the available genomes of *L. murinus* ranging from 96.18 to 97.99% and 96.14 to 98.06%, respectively, which were values that would undoubtedly classify them into the species *L. murinus* (Additional file 9).

The genomes of CR141 and CR147 each consisted of a single circular chromosome of 2,288,482 and 2,290,452 bp, respectively, both with a G + C content of 39.86% and no detected plasmids (Additional file 10). The general genomic features are shown in Additional file 11. In the clusters of orthologous groups (COG) assignment, most functional category genes were shared by the two genomes (Fig. 5b). The three exceptions were the ‘Transcription’, ‘Replication, recombination and repair’ and ‘Mobilome: prophages, transposases’ categories due to more copies of genes coding for phage-related proteins and transposases in the CR147 genome. At the nucleotide level, alignment of the whole genome sequences of CR141 and CR147 revealed relatively high sequence conservation, which was consistent with the ANI value of 99.7% shared by them, but there existed a 1.6-Mb chromosomal inversion centred around the replication terminus region (Fig. 5c). Additionally, 40 and 46 coding sequences (CDSs) were identified to be unique in the CR141 and CR147 genomes, respectively (Additional file 12). Most of these genes were functionally unannotated (23 in CR141 and 31 in CR147), but some genes with predicted functions were present. These strain-specific CDSs were intensively distributed in some regions, which was evident from the genome alignment, leaving a gap where the two genomes lacked sequence homology (Fig. 5c). Although the two genomes share high sequence similarity and a conserved genetic background, the chromosomal rearrangement and strain-specific genes may lead to different physiological properties.

### *L. murinus* strain CR147 exhibits CR-related health-promoting effects in an in vitro model and in *C. elegans*

**Fig. 6:**
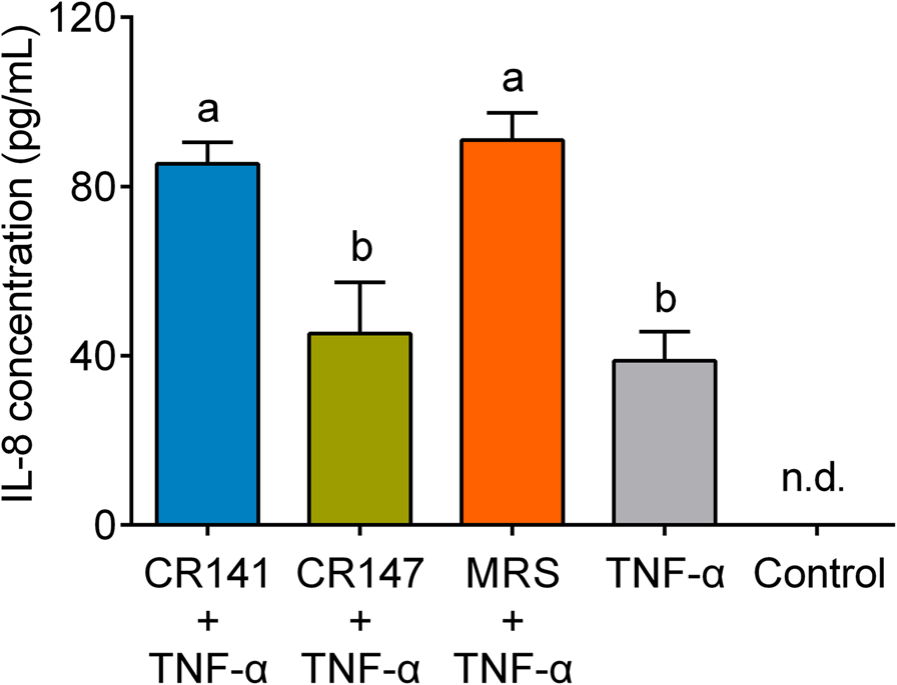
Bacterial culture supernatant (BCS) from *L. murinus* CR147 exerts anti-inflammatory effects on Caco-2 cells. Caco-2 cells were stimulated with TNF-α at 10 ng/mL and simultaneously treated with BCS from *L. murinus* or sterile MRS medium (10%, *v*/*v*) for 6 h. The levels of secreted IL-8 in the culture supernatant were measured by ELISAs. Control, negative control without TNF-α. Data are shown as the mean ± s.e.m. *n* = 4 for all groups. Values of each group with the same letters are not significantly different by one-way analysis of variance (ANOVA) followed by Tukey’s post hoc test. n.d., non-detectable

To determine whether key members in the CR-modulated gut microbiota contribute to the reduction of inflammation, we used TNF-α-stimulated Caco-2 cells as an in vitro model of inflammation and tested IL-8 production to evaluate the anti-inflammatory properties of these CR-enriched *L. murinus* strains CR141 and CR147. IL-8, which can be produced by TNF-α-simulated intestinal epithelial cells, is an important mediator of inflammation that recruits neutrophils into the inflamed tissue. As shown in Fig. 6, treatment with TNF-α did upregulate IL-8 production compared with the negative control without TNF-α. Caco-2 cells were then incubated with bacterial culture supernatant (BCS) or sterile MRS medium to examine their effects on IL-8 secretion. BCS from *L. murinus* CR147, but not that from CR141, significantly reduced IL-8 secretion compared with the MRS medium, implying that some soluble substances secreted by *L. murinus* CR147 inhibited IL-8 production.

**Fig. 7:**
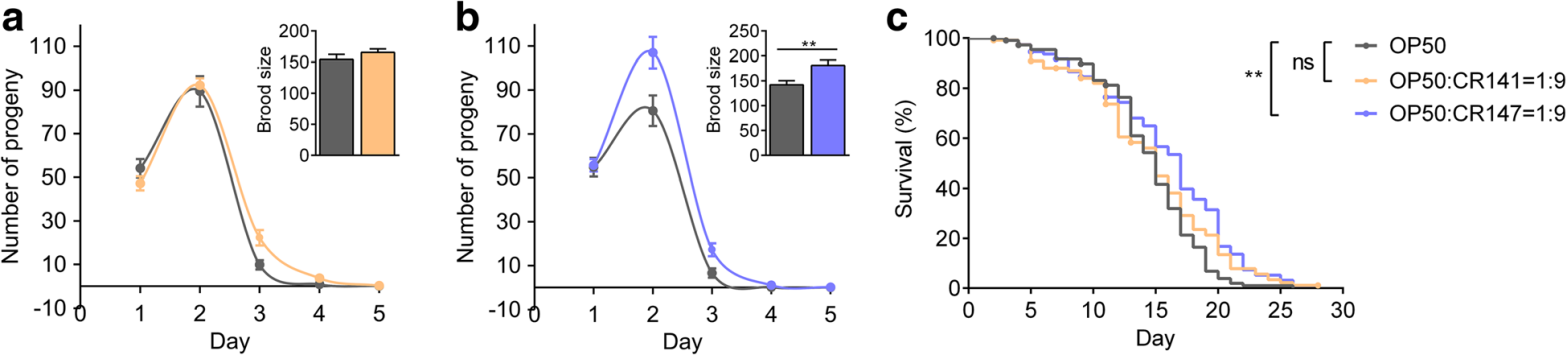
*L. murinus* CR147 increases the brood sizes and lifespan of *C. elegans* without changing egg-laying schedules. Worms were synchronized at L4-stage larvae at day 0. The egg-laying schedules and brood sizes of the worms fed a 1:9 mixture of *E. coli* OP50 and **a** *L. murinus* CR141 (OP50, *n* = 15; OP50:CR141 = 1:9, *n* = 14) or **b** *L. murinus* CR147 (OP50, *n* = 14; OP50:CR147 = 1:9, *n* = 15). Data are shown as the mean ± s.e.m. **c** Survival curves of *C. elegans* fed a 1:9 mixture of OP50 and *L. murinus* compared with the lifespan of the worms fed OP50 alone (OP50, *n* = 111; OP50:CR141 = 1:9, *n* = 116; OP50:CR147 = 1:9, *n* = 119). Each mNGM plate contained 10 mg of bacteria (wet weight). Differences were assessed by unpaired *t* test (two-tailed) (**a**, **b**) or log-rank test (**c**). ns, no significant difference; **P < 0.01 vs OP50 group (control). *n* indicates the number of worms per group

Next, we used the *C. elegans* model to determine whether the *L. murinus* CR141 and CR147 strains, which were predominant in the CR mice gut, had the potential to promote host health and longevity. *C. elegans* has a short and reproducible lifespan and can be easily maintained in monoxenic culture, i.e., with single bacterial strain, in laboratory conditions, making it an excellent model to investigate the role of microbes in host health and ageing [20]. Considering that worms fed only *L. murinus* CR141 or CR147 had much smaller body sizes and brood sizes (data not shown), we tested the influence of *L. murinus* CR141 or CR147 on the reproduction and lifespan of worms in combination with *Escherichia coli* OP50, standard food for *C. elegans* in the laboratory. Synchronized L4-stage larvae were transferred to plates containing a mixture of *E. coli* OP50 and *L. murinus* CR141 or CR147 at various ratios (9:1, 1:1 and 1:9). According to the previous report, when some bacteria were used as the food instead of *E. coli* OP50, *C. elegans* showed extended lifespan as well as delayed development because these bacteria reduced the food intake of the worms [21]. Then, we first confirmed that none of these conditions affected the time taken for worms to reach reproductive age (Fig. 7a, b; Additional file 13a, b), indicating that the developmental rate of the worms was not affected by these *Lactobacillus* strains. Interestingly, the 1:9 mixture of OP50 and CR147 significantly increased the brood sizes of the worms (Fig. 7b). Moreover, lifespan assays indicated that the lifespan of worms fed a 1:9 mixture of OP50 and CR147 was significantly prolonged compared with that of worms fed the OP50 control, while a 1:9 mixture of OP50 and CR141 had no lifespan-extending effect on the worms (Fig. 7c). However, when the proportion of CR147 in the mixture was reduced to 10%, the lifespan of worms was not extended (Additional file 13c), suggesting that CR147 must be in high abundance to exert its beneficial effects.

Taken together, although *L. murinus* CR141 and CR147 have highly similar genome sequences, only strain CR147 showed health-promoting effects both in vitro and in vivo.

### Administration of *L. murinus* CR147 reduces intestinal permeability and systemic inflammatory marker in old microbiota-colonized mice

To investigate the effect of *L. murinus* CR147 in a mouse model, we colonized 10-month-old germ-free mice with the microbiota from 18-month-old SPF mice (old microbiota, OM) with or without additional *L. murinus* CR147 supplementation and tested their gut barrier function and circulating levels of TNF-α. By 16S rRNA gene sequencing, we showed that the relative abundance of *Lactobacillus* was much higher in the OM + CR147 group mice than in the OM group (1.08 versus 0.18%, respectively; *P* < 0.001, Mann-Whitney *U* test; Additional file 14).

**Fig. 8:**
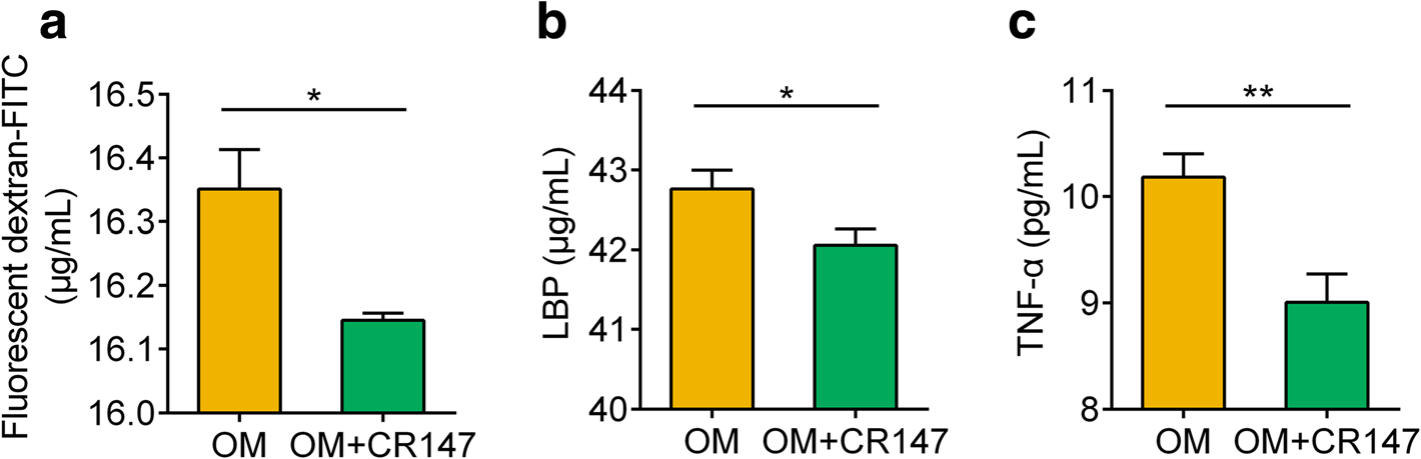
*L. murinus* CR147 reduces intestinal permeability and systemic inflammatory marker in old microbiota-colonized gnotobiotic mice. **a** Intestinal permeability of mice colonized with old microbiota (OM) or OM plus *L. murinus* CR147 (OM + CR147) measured by FITC-dextran translocation to systemic circulation following oral gavage (OM, *n* = 7; OM + CR147, *n* = 6). **b** Serum LBP (OM, *n* = 7; OM + CR147, *n* = 6). **c** Serum TNF-α (*n* = 5 per group). All data are shown as the mean ± s.e.m. Unpaired *t* test (two-tailed) was used to analyse the variation between the two groups. *P < 0.05 and **P < 0.01

In vivo intestinal permeability was measured by performing oral gavages with 4000 Da fluorescein isothiocyanate (FITC)-labelled dextran, followed by measurement of the translocation of fluorescence into the plasma. Fourteen days after inoculation, the mice additionally administered *L. murinus* CR147 exhibited significant decreases in plasma DX-4000-FITC concentrations compared with the OM group mice (Fig. 8a), indicating that *L. murinus* CR147 reduced intestinal permeability. Consistent with the in vivo assessment of intestinal permeability, the serum levels of LBP were significantly lower in the OM + CR147 group than in the OM group (Fig. 8b). Moreover, the OM + CR147 group mice had lower levels of TNF-α (Fig. 8c). These results indicated that *L. murinus* CR147 enhanced intestinal barrier function and subsequently reduced the translocation of bacterial products, reducing systemic inflammatory marker in old microbiota-colonized mice.

## Discussion

Chronic inflammation is a strong risk factor for both morbidity and mortality in elderly people [22, 23], and ageing-associated microbial dysbiosis may drive this pathological process [16, 24]. Attenuation of inflammation is one of the most important ways of CR to improve health during ageing [25, 26]. Previously, we showed that life-long CR mice had a *Lactobacillus*-predominated gut microbiota [2]. In the current work, we showed that this *Lactobacillus*-predominated microbial community was rapidly attained in mice from the very early stage of CR, i.e., within 2 weeks. Data obtained with various experimental systems showed that a *Lactobacillus murinus* strain isolated from the feces of CR mice was one of the key members contributing to the protection of gut barrier and the attenuation of chronic systemic inflammation.

Diet-related changes of the gut microbiota have been broadly investigated and are considered pivotal mediators in the pathogenesis and development of metabolic diseases, such as obesity and diabetes [14, 27–29]. The major aspect by which diet influences the gut microbiota is its contents, namely, the macro- and micro-nutrients of the consumed meals [28, 30–32]. Accumulating evidence has shown that the quantities of food consumed could also modulate the gut microbiota [2–4], but previous studies provided the correlative relationships that these changes in the gut microbiota may impact or modulate the beneficial effects associated with CR. For example, our life-long mouse study showed that *Lactobacillus* spp., which were dramatically enriched by CR in midlife, were strongly positively correlated with lifespan and negatively correlated with antigen load from the gut microbiota [2]. In the current work, we isolated the predominant beneficial bacterium in the CR mice gut and validated its capacity to extend the lifespan of *C. elegans* and to be anti-inflammatory in Caco-2 cells and gnotobiotic mice. These findings provide causative evidence of the role of the gut microbiota in the beneficial effects associated with CR.

Emerging evidence suggests that homeostasis of the gut microbiota is tightly linked to the health and even the ageing process of their host [33, 34]. With invertebrate model systems, such as the fruit fly *Drosophila melanogaster* and the nematode *C. elegans*, scientists have demonstrated that some bacterial variants serendipitously determined the host lifespan [35, 36] and small molecules secreted by the bacteria have been linked to host longevity in the context of specific bacterial backgrounds [37, 38]. However, in mammals, identifying the specific members involved in driving or slowing the ageing process from a complex gut microbial community and determining their mechanisms of action is still very challenging. Numerous studies have shown characteristic changes in gut microbial composition in elderly people [39–41], and manipulation of the gut microbiota with diet or probiotics/prebiotics has been shown to improve their health [42, 43]. A recent study in a mouse model suggested that the ageing-associated microbiota promoted gut permeability and systemic inflammation, but the key members of the microbial community that altered barrier function with age were not identified [16]. Most of these studies used taxon-based analysis to investigate the relationships between the microbiota and ageing, which often produce inconsistent or conflicting results. One of the reasons is that bacterial functions are often strain-specific. For example, multiple *Bifidobacterium pseudocatenulatum* strains isolated from the same habitat have differential responses to dietary intervention as well as strain-specific correlations with clinical parameters of the host [44]. In the current study, we showed that two strains of *Lactobacillus murinus* with highly similar genomic sequences, which were both isolated from the CR mice gut, had different influences on their hosts. Only the strain *L. murinus* CR147 had the capacity to prolong the lifespan and increase the brood sizes of the worms to downregulate the IL-8 produced by TNF-α-stimulated Caco-2 cells and to improve gut barrier function and reduce systemic inflammatory markers in the old gut microbiota-colonized gnotobiotic mice. Our results highlight that we need to identify the gut microbes that contribute to host health phenotypes at the strain level and confirm their functions using model systems in which the molecular mechanisms can be elucidated.

The modes by which probiotics exert their beneficial effects on hosts can be classified into three main categories: modulating the intestinal microbiota, enhancing the intestinal barrier function and regulating the mucosal immune system [45]. *Lactobacillus* is one of the genera that are most commonly used as probiotics, and members of this genus have been shown to be anti-inflammatory and protective of gut barrier function. Surface-layer proteins (Slps) derived from the probiotic strain *Lactobacillus helveticus* R0052 was found to block the adherence of enterohaemorrhagic *Escherichia coli* O157:H7 to epithelial cells and to attenuate pathogen-induced changes in epithelial barrier function in vitro [46]. A soluble protein, P40, secreted by the well-documented probiotic *Lactobacillus rhamnosus* GG (LGG) ameliorated DSS- and oxazolone-induced colitis through the activation of epidermal growth factor receptor (EGFR) [47]. Both VSL#3-derived *Lactobacillus paracasei* and human-derived *Lactobacillus casei* express a *prtP*-encoded serine protease lactocepin, which mediated their anti-inflammatory effects by selectively degrading a pro-inflammatory chemokine [48]. The *L. murinus*, the same species found in our work, could promote regulatory T cell development and suppress the development of DSS-induced colitis in mice [49]. Moreover, a recent study reported that the metabolism of tryptophan into indole metabolites by *L. murinus* contributed to the gut immune homeostasis of the host through the suppression of pro-inflammatory T helper 17 cells in the gut mucosa [50]. Further experiments need to be performed to determine the specific metabolites or cell components that mediate the health benefits of *L. murinus* CR147.

## Conclusions

In 1907, Elie Metchnikoff proposed the theory that senility is due to the poisoning of the body by the products of certain members of the human gut microbiota, and he had great faith that manipulation of the intestinal microbiota would extend lifespan [51]. Increasing experimental evidence has begun to support his hypothesis. Our study demonstrates that the predominant *L. murinus* strain isolated from the feces of CR mice is one of the key members of the microbiota that contributes to the beneficial effects of CR in mammalian hosts. Such key beneficial bacteria may become an attractive target for mitigating age-associated inflammation and elongating lifespan in humans.

## Methods

### Animal calorie restriction intervention

Specific pathogen-free (SPF), 6-week-old male C57BL/6J mice were purchased from SLAC Inc. (Shanghai, China). The mice arrived at the animal center of Shanghai Jiao Tong University at the same time and maintained under a 12-h dark/light cycle (lights on at 6:30 AM) at a temperature of 22 °C ± 3 °C in accredited animal facilities. All the animal experimental procedures were approved by the Institutional Animal Care and Use Committee of the School of Life Sciences and Biotechnology, Shanghai Jiao Tong University (No. 2014005). Before the start of the experiment, mice were maintained on a normal chow diet (3.52 total kcal/g; P1101F, SLAC Inc., Shanghai, China) for 2 weeks. Then, the mice were randomly assigned into two groups (10 mice per group), fed ad libitum with a normal chow diet (NC group) or fed 70% of the ad libitum (CR group). The daily food consumption in the NC group was recorded over 1 week and averaged to determine the amount of food for the CR group in the following week. All mice were housed individually during the study to avoid any competition effect to ensure each mouse in the CR group can get 70% of the NC group [17].

During the trial, body weight was measured weekly. Fresh feces were collected daily and stored at − 80 °C. Oral glucose tolerance test (OGTT) was conducted as follows: after 5 h of food deprivation, mice were administrated by oral gavage with glucose at a dose of 2.0 g/kg body weight, and blood glucose concentrations were determined in blood collected from the tip of tail vein before and 15, 30, 60, 90 and 120 min after glucose administration with a blood glucose meter (ACCU-CHEK® Performa, Roche, USA). Blood samples were collected after fasting for 6 h, and serum was isolated through centrifugation at 4000 rpm at 4 °C for 15 min and stored at − 80 °C. White adipose tissues (epididymal, mesenteric, subcutaneous inguinal and retroperitoneal) and vastus lateralis muscles were collected and weighed after mice were sacrificed by cervical dislocation.

Enzyme-linked immunosorbent assays (ELISAs) were conducted according to the manufacturer’s instructions. The ELISA kits for serum fasting insulin and lipopolysaccharide (LPS)-binding protein (LBP) were from Mercodia Inc. (10-1249-01; Uppsala, Sweden) and Cell Sciences, Inc. (CKM043; Canton, MA, USA), respectively. The kits for leptin, adiponectin and tumor necrosis factor-alpha (TNF-α) were all purchased from R&D Systems, Inc. (MOB00, MRP300, MHSTA50; Minneapolis, MN, USA). Serum LBP was determined after a dilution of 1:800. All the ELISA kits used in the current study are highly sensitive kits. According to the instructions of the kits, the detection limit is 1 ng/mL for LBP, 0.025 ng/mL for insulin, 22 pg/mL for leptin, 0.007 ng/mL for adiponectin and 0.295 pg/mL for TNF-α.

Statistical analysis was carried out by GraphPad Prism version 6.01 (GraphPad Software, Inc.). Unpaired *t* test (two-tailed) or Mann-Whitney *U* test (two-tailed) was used to analyse variation between the NC and CR groups, depending on data distribution. Differences were considered statistically significant when *P* < 0.05.

### 16S rRNA gene V3-V4 region sequencing and data analysis

DNA extraction from all fecal matters was conducted as previously described [52]. Sequencing library of the V3-V4 region of 16S rRNA gene was constructed according to the manufacturer’s instructions (Part # 15044223 Rev. B; Illumina Inc., USA) with some modifications as previously described [53] and sequenced on the Illumina MiSeq platform (Illumina Inc., USA). Quality control of raw data was conducted as previously described [53]. Unique sequences were obtained from quality-filtered sequences through dereplication and sorted in descending order of abundance, and singletons were discarded. Then, unique sequences were clustered into OTUs using UPARSE algorithm [54] with 97% similarity, during which the most abundant unique sequence of each cluster was selected as representative sequence. All the representative sequences were subjected to further reference-based chimera detection with UCHIME algorithm [55] against Ribosomal Database Project (RDP) training database (v9) [56]. The high-quality sequences were classified into their corresponding OTUs using Usearch global alignment algorithm [57] at a 97% cutoff of identity. Representative sequences for all OTUs were built into a phylogenetic tree using FastTree [58] and subjected to RDP Classifier [59] for taxonomic assignment with a bootstrap cutoff of 80%.

The sequences of each sample were subsampled to 8000 (1000 permutations) to even the difference in sequencing depth among samples for further analysis. Three samples were excluded from further analysis because their high-quality reads were less than 8000. Richness and diversity of microbiota in each sample were assessed with observed OTUs and Shannon diversity index calculated on QIIME platform (version 1.8.0) [60], respectively. Principal coordinate analysis (PCoA) was performed to illustrate the dynamic alteration of gut microbiota in response to calorie restriction by QIIME. The statistical significance between different groups was analyzed with multivariate analysis of variance (MANOVA) in MATLAB R2014a (The MathWorks Inc., Natick, MA, USA). Both PCoA and MANOVA clustering analysis were calculated with Bray-Curtis distances based on OTU abundance data.

Random forests [61] were used to establish the classification model between the NC and CR groups. Samples of the day 84 from the NC and CR groups were included for discrimination using the ‘randomForest’ package in R [62] with 1000 trees and all default settings. The generalization error of the classifier was estimated using leave-one-out cross-validation. According to the increase in error by removing a specific OTU from the set of predictors, each OTU was assigned a score of mean decrease in accuracy (MDIA) as its importance score. All OTUs were ranked by their importance scores, and the OTUs with an importance score of MDIA at least 0.001 were considered highly predictive to separate gut microbiota between the NC and CR groups.

The correlation coefficient between individual OTU were determined using the method for repeated observations described by Bland and Altman [63] based on their abundances in the CR group of the first 2 weeks. Then, correlations among the 50 key OTUs were visualized as a network in Cytoscape v3.2.0 [64]. The correlations were converted to a correlation distance (1-correlation coefficient) and then clustered using the Ward clustering algorithm in MATLAB R2014a. From the top of the hierarchical clustering tree, we used permutational multivariate analysis of variance (PerMANOVA, 9999 permutations, *P* < 0.001) to sequentially determine whether the two clades were significantly different to cluster the OTUs into co-abundance groups (CAGs). PerMANOVA was computed using ‘vegan’ package version 2.0-10 in R [65].

### Sequence-guided isolation of predominant *Lactobacillus* spp.

We isolated the predominant *Lactobacillus* spp. in CR mice gut via a ‘sequence-guided isolation’ scheme as follows. (1) Fresh feces were collected from five mice after 2 weeks of CR, transported into an anaerobic chamber (A45, Don Whitley Scientific, UK) and pooled together. (2) Fecal matters were mixed and tenfold serially diluted with sterile anaerobic phosphate-buffered saline (PBS) (pH 7.2; containing l-cysteine at 0.1%). The 10^−4^ dilutions were spread onto MRS agar plates (Qingdao Hope Bio-Technology Co., Ltd., China; containing l-cysteine at 0.05% and CaCO_3_ at 0.4%) and incubated at 37 °C under anaerobic conditions for 48 h. Based on the colony morphology of *Lactobacillus* and the calcium-dissolving zone, 250 colonies were selected. (3) All the isolates were tested by *Lactobacillus* group-specific PCR with primers Lac1 (5′-AGCAGTAGGGAATCTTCCA-3′) and Lac2 (5′-ATTYCACCGCTACACATG-3′) [66], and a total of 217 isolates were identified as *Lactobacillus* spp.. (4) Enterobacterial Repetitive Intergenic Consensus (ERIC) sequences of the genome from these 217 isolates were amplified by PCR with primers ERIC1 (5′-ATGTAAGCTCCTGGGGATTCAC-3′) and ERIC2 (5′-AAGTAAGTGACTGGGGTGAGCG-3′) [67]. The isolates were considered as the same *Lactobacillus* strain if they shared the same ERIC-PCR profiles. These 217 isolates were classified into ten strains (E1-E10) (Additional file 7a). For each strain, one isolate was randomly selected as the representative. (5) The total DNA was extracted from the fresh feces collected from two mice (M5 and M7) after 2 weeks of CR. The V3 regions in 16S rRNA gene of the mice fecal DNA were amplified with universal bacterial primers P2 (5′-ATTACCGCGGCTGCTGG-3′) and P3 (5′-CGCCCGCCGCGCGCGGCGGGCGGGGCGGGGGCACGGGGGGCCTACGGGAGGCAGCAG-3′) [68]. In the denaturing gradient gel electrophoresis (DGGE) fingerprinting of V3 region in 16S rRNA gene from mice feces, the most dominant band shared 100% sequence similarity with OTU1 (the most predominant *Lactobacillus* phylotype from the high-throughput sequencing data). V3 region in 16S rRNA gene of the genomic DNA from the aforementioned ten isolated strains were also amplified. In the DGGE profiles, the strains CR141 and CR147 (representative of E1 and E2, respectively) migrated to the identical position as the most dominant band of the fecal DNA fingerprint of CR mice. The strains CR141 and CR147 could be considered as the representatives of the most abundant *Lactobacillus* spp. in the CR mice gut (Additional file 7b) and were further identified by 16S rRNA gene sequencing.

### Whole-genome sequencing and analysis of *Lactobacillus* spp.

Whole-genome DNA of the two strains of *Lactobacillus* isolates (named CR141 and CR147) were extracted with a blood and cell culture DNA kit (13,323, Qiagen, USA). Then, the genomes of CR141 and CR147 were sequenced on a PacBio RS II platform with a 20-kb library and P6-C4 sequencing chemistry, generating approximately 309-fold and 245-fold coverage, respectively (Nextomics Biosciences, Wuhan, China). The subreads of each strain were de novo-assembled into one contig, corresponding to its chromosome, with HGAP version 2.3.0 pipeline [69]. Protein-coding sequences (CDSs) were predicted with Prodigal v2.6.0 [70]. tRNAs and rRNAs were predicted with tRNAscan-SE v1.23 [71] and RNAmmer v1.2 [72], respectively. The identified CDSs were searched against the NCBI non-redundant protein database with BLASTP. The Clusters of Orthologous Groups (COG) assignment for all CDSs was conducted using COGtriangles in COGsoft (ftp://ftp.ncbi.nih.gov/pub/wolf/COGs/COGsoft/).

Genome sequences of the phylogenetic relatives were retrieved from the NCBI database. Sequences were uploaded into the Jspecies V1.2.1 [19] to calculate the average nucleotide identity (ANI) values with default parameters. ANI has been demonstrated to substitute DNA-DNA hybridization (DDH) to circumscribe species at genomic level.

At the nucleotide level, pairwise genome alignment of the two strains of *Lactobacillus* spp. was generated with WebACT (BLASTN with default parameters) [73] and visualized with the Artemis Comparison Tool (ACT, score cutoff with 2000) [74]. For the analysis, the replication origin of each genome was located with the web-based system Ori-Finder [75]. Strain-specific genes were identified with mGenomeSubtractor [76] and defined with *H* value < 0.42. Prophage-related regions, genomic island (GI)-like regions and transposases were identified with VRprofile [77].

### In vitro anti-inflammatory capacity test of *Lactobacillus* spp.

*Lactobacillus* isolates were anaerobically cultured in de Man, Rogosa, and Sharp (MRS) broth (Qingdao Hope Bio-Technology Co., Ltd., China) at 37 °C for 12 h, and then, bacterial culture was diluted with MRS broth to a density of 1.0 absorbance units at a wavelength of 670 nm. The bacterial culture supernatant (BCS) was separated from bacterial cells through centrifugation at 5000×*g* for 10 min, filtered through a 0.22-μm filter (Millipore Co., Cork, Ireland) and stored at − 80 °C until use.

The human colon adenocarcinoma Caco-2 cell lines were purchased from the Cell Bank of Type Culture Collection of Chinese Academy of Sciences (Shanghai, China) and maintained in Dulbecco’s modified Eagle’s medium (DMEM; SH30243.01B, Hyclone, USA) supplemented with 10% fetal bovine serum (FBS; SH30084.03B, Hyclone, USA), penicillin (100 units/mL) and streptomycin (100 μg/mL) at 37 °C in a humidified incubator under 5% CO_2_/air atmosphere. Cells were seeded into 24-well tissue culture plates at a density of 1 × 10^5^ cells/well in 1 mL DMEM. Media were changed every other day. Experiments were initiated on day 7 after seeding, within 5 days after confluency. Cell culture medium was changed to FBS-free culture medium 24 h prior to experiments. Caco-2 monolayers were rinsed with PBS (SH30256.01B, Hyclone, USA) once and then treated with human recombinant human TNF-α (10 ng/mL; 300-01A, PEPROTECH, USA) and bacterial BCS at 10% simultaneously for 6 h. After incubation, culture medium were collected and centrifuged at 4000×*g* for 5 min. The cell experiment performed twice with four repetitions of each sample. Then, the supernatant was collected for determination of pro-inflammatory chemokine IL-8 using ELISA kit (D8000C, R&D systems, Minneapolis, MN, USA). According to the protocol of the kits, the detection limit is 7.5 pg/mL.

### *C. elegans* lifespan and reproduction analysis

*C. elegans* Bristol strain N2 used in this study was provided by the Caenorhabditis Genetics Center, which is funded by NIH Office of Research Infrastructure Programs (P40 OD010440). Nematodes were maintained and propagated on nematode growth medium (NGM) according to techniques as described [78]. *Escherichia coli* OP50 [79] was used as the standard feed for nematode cultivation and was cultured in LB medium. Isolates of *Lactobacillus* spp. were used as test food sources for worms and were cultured using de Man, Rogosa, and Sharp broth (MRS broth; Qingdao Hope Bio-Technology Co., Ltd., China). Cultured bacteria were washed twice and resuspended with worm M9 buffer [78]. Then, *E. coli* OP50 was mixed with isolates of *Lactobacillus* spp. at various ratios and 100 μL of the resulting bacterial suspension (10 mg/100 μL) was spread on peptone-free modified NGM (mNGM) in 6.0-cm-diameter plates to feed worms. During the trial, worms were all incubated at 25 °C.

Synchronized L4-stage larvae were obtained as the timed hatch-off method described previously [80] with minor modifications. Briefly, to remove all possible contaminants, eggs were recovered from adult worms after exposure to sodium hypochlorite/sodium hydroxide solution and then placed onto the standard nematode growth medium (NGM) plates covered with *E. coli* OP50. Plates were incubated for about 2.5 days to allow worms to grow to gravid adults. About ten young, gravid worms (per plate) were transferred onto new NGM plates with *E. coli* OP50 and allowed to lay eggs for 4 h. Then, the plates were incubated for about 40 h to allow the worms to develop into L4-stage larvae. Both lifespan and reproduction assays were initiated with synchronized L4-stage worms.

The lifespan of worms was determined as described previously [78], with minor modifications. Briefly, 20–30 healthy L4 larvae were transferred to at least five mNGM plates covered with lawns of each test food source, and the plates were incubated at 25 °C. The numbers of live, dead and censored worms were scored daily. To avoid confusing the original worms with their progeny, worms were transferred daily to fresh mNGM plates for 5 days until their progeny production ceased. Then, worms were transferred to fresh mNGM plates every 4th day. A worm was considered dead when it failed to respond to a gentle touch with a worm picker. Worms were censored if they were lost, had crawled off the plates, had burrowed into the medium, undergone matricidal hatching or exhibited physical abnormalities such as protruding vulva. All lifespan assays were performed at the same time with more than 100 animals per group. Nematode survival curves were plotted using the Kaplan-Meier method, and lifespan differences between groups were evaluated by log-rank test on OASIS 2 platform (Online Application for Survival Analysis 2) [81]. Differences were considered statistically significant when *P* < 0.05.

Reproduction assays were performed as previously described [80]. Briefly, one L4 hermaphrodite was transferred to per mNGM plate and 15 plates were set up per condition. Then, the plates were incubated at 25 °C. The parental worms were transferred every 24 h onto fresh mNGM plates until the last worm ceased reproduction. The progeny were left to develop for 2 days, and the progeny number was then determined. Worms were also censored as described above. Reproduction assays for strains CR141 and CR147 were performed in two separate experiments with more than ten worms.

### Gut microbiota transplantation

Fresh fecal matters were collected from a specific pathogen-free (SPF), 18-month-old male C57BL/6J mice and transported into an anaerobic workstation (A45, DWS, UK). Fecal suspension was prepared as previously described [82]. Briefly, fecal samples were diluted to 20 μg/mL with sterile Ringer buffer (containing 9 g of sodium chloride, 0.4 g of potassium chloride, 0.25 g of calcium chloride dihydrate and 0.05% l-cysteine-HCl in 1 L buffer). Homogenized suspension was prepared by thorough vortex for 5 min, and then, it was settled by gravity for 5 min. The clarified supernatant was transferred to new tubes, and an equal volume of 20% skim milk (LP0031, Oxoid, UK) was added. The fecal suspension, old microbiota (OM), was preserved at − 80 °C until use. The strain *L. murinus* CR147 was cultured in MRS broth and was then washed twice and resuspended with Ringer buffer, and an equal volume of 20% skim milk was added, reaching a density of 10^9^ cells/mL. Bacterial suspension for *L. murinus* CR147 was also stored at − 80 °C until use.

Seven males and eight females of germ-free (GF) C57BL/6J mice at the age of 10 months were purchased from and maintained at the Laboratory Animal Center of the Third Military Medical University, Chongqing, China. All experimental procedures were approved by the Institutional Animal Care and Use Committee of the Third Military Medical University, Chongqing, China. The old germ-free mice (10-month-old) were difficult to obtain, and the animal trail was performed once. Mice were housed in flexible-film plastic isolators under a regular 12-h dark/light cycle (lights on at 06:00 AM) and fed with a sterile normal chow diet ad libitum. Surveillance for bacterial contamination was performed by a periodic bacteriologic examination of feces, food and padding.

Before the experiment, the animals were randomly assigned into two isolators for acclimatization, and in the same isolator, males and females were separately caged. One week later, four male and four female mice in one isolator were administrated by oral gavage with 200 μL fecal suspension of old microbiota (OM group), and three male and four female mice in another isolator were inoculated with 100 μL fecal suspension and 100 μL bacterial suspension of *L. murinus* CR147 (OM + CR147 group). A repeat inoculation was performed the next day to reinforce the microbiota transplantation.

Fourteen days after the first inoculation, fresh feces were collected for gut microbiota analysis. Intestinal permeability in vivo was measured based on the appearance in blood of 4000 Da fluorescent dextran-FITC (DX-4000-FITC) (FD4000; Sigma-Aldrich, St. Louis, Missouri, USA) administrated by oral gavage as previously described [15]. Briefly, after fasting for 6 h, mice were inoculated by oral gavage with DX-4000-FITC (500 mg/kg body weight, 125 mg/mL). After 4 h, 120 μL of blood was collected from the tip of tail vein and plasma was separated through centrifugation at 4 °C, 12000×*g* for 3 min. Plasma was diluted in an equal volume of PBS (pH 7.4) and analyzed for DX-4000-FITC concentration with a fluorescence spectrophotometer (HTS-7000 Plus-plate-reader; Perkin Elmer, Wellesley, Massachusetts, USA) at an excitation wavelength of 485 nm and emission wavelength of 535 nm. A standard curve was obtained by diluting FITC-dextran in non-treated plasma diluted with PBS (1:3 *v*/*v*). Then, whole blood samples were collected from the orbital plexus, and serum was isolated for LBP and TNF-α testing.

## Additional files

Additional file 1: Supplementary results about summary of sequencing on the V3-V4 region of 16S rRNA gene. (DOCX 13 kb)

Additional file 2: Alpha diversity of the gut microbiota during short-term calorie restriction. a Observed OTUs and b Shannon diversity index at sampling level of 8000. Data are shown as mean ± s.e.m. Mann-Whitney *U* test (two-tailed) was used to analyse variation between the NC and CR groups at the same time point. *P < 0.05 and ***P < 0.001 vs the NC group. Sample sizes are the same as in Fig. 2. (TIFF 295 kb)

Additional file 3: The taxonomy, co-abundance group and dynamics of the 50 key OTUs. (XLSX 13 kb)

Additional file 4: Alpha diversity of the gut microbiota in the first 14 days of calorie restriction. a Observed OTUs and b Shannon diversity index at sampling level of 8000. Data are shown as mean ± s.e.m. Mann-Whitney *U* test (two-tailed) was used to analyse the variation between the NC and CR groups at the same time point. *P < 0.05, **P < 0.01 and ***P < 0.001 vs the NC group. Sample sizes are the same as in Fig. 3. (TIFF 317 kb)

Additional file 5: Impact of calorie restriction on gut microbiota in the first 14 days. a PerMANOVA test (9999 permutations) based on Bray-Curtis distances of samples. (XLSX 8 kb)

Additional file 6: Dynamics of OTU1 in CR group during the first 14 days of calorie restriction. Data are shown as mean ± s.e.m. *N* was the day when OTU1 began to increase, as determined by its absolute value of logarithmic (base 2) fold change in relative abundance greater than 1 (|log_2_-fold change| > 1), and became the most abundant phylotype in CR mice. Kruskal-Wallis test followed by Dunn’s multiple comparison test was used to analyse the variation relative to day 0. *P < 0.05, **P < 0.01 and ***P < 0.001 vs day 0. (TIFF 143 kb)

Additional file 7: Sequence-guided isolation of the predominant *Lactobacillus* spp. in CR mice gut. a 217 *Lactobacillus* isolates were classified into ten strains (E1-E10) based on ERIC-PCR profiles. M, DNA ladder. b Strains CR141 (E1) and CR147 (E2) were identified as representatives of the most abundant *Lactobacillus* spp. based on their co-migration pattern with the most dominant band in the fecal DNA fingerprint of CR mice in the DGGE profiles. M5 and M7, fecal DNA samples of mice subjected to CR for 2 weeks. NC, negative control. c/d Electron micrograph of strain CR141 (c) and CR147 (d). e The growth curves of CR141 and CR147 in the MRS medium. f Changes of the pH value during the growth of CR141 and CR147 in the MRS medium. (TIFF 4862 kb)

Additional file 8: The 16S rRNA gene sequence similarity between the two strains of *Lactobacillus* spp. and their close relatives. (XLSX 9 kb)

Additional file 9: ANI values between the two strains of *Lactobacillus* spp. and their phylogenetic relatives. (XLSX 9 kb)

Additional file 10: Genome atlas of the two strains of *Lactobacillus murinus*. a *L. murinus* CR141. b *L. murinus* CR147. From inner to outer: GC skew (G − C)/(G + C), mean centered GC content (red-above mean, blue-below mean), tRNAs/rRNAs, CDS (reverse and forward strand), m4C and m6A sites in CDS/rRNA/tRNA (reverse and forward strand), m4C and m6A sites in inter-gene regions. (TIFF 1217 kb)

Additional file 11: Major genomic features of strains CR141 and CR147. (XLSX 9 kb)

Additional file 12: Strain-specific CDSs determined by pairwise comparison. a The 40 CR141-specific CDSs as determined by comparison with CR147. b The 46 CR147-specific CDSs as determined by comparison with CR141. (XLSX 12 kb)

Additional file 13: Effects of *L. murinus* on the egg-laying schedules, brood size and lifespan of *C. elegans*. Worms were synchronized L4-stage larvae at day 0. The egg-laying schedules and brood sizes of worms fed 9:1 or 1:1 mixture of *E. coli* OP50 and a *L. murinus* CR141 or b *L. murinus* CR147. Data are shown as mean ± s.e.m. c Survival curves of *C. elegans* fed a 9:1 mixture of *E. coli* OP50 and *L. murinus* compared with the lifespan of the worms fed OP50 alone. Each mNGM plate contained 10 mg of bacteria (wet weight). Differences were assessed by unpaired *t* test (two-tailed) (a, b) or log-rank test (c). *N* indicates the number of worms per group. (TIFF 646 kb)

Additional file 14: *L. murinus* CR147 supplementation increases the abundance of *Lactobacillus* in old microbiota-colonized gnotobiotic mice gut. After 14 days of the inoculation, the abundance of *Lactobacillus* in fecal microbiota of mice colonized with old microbiota (OM group) or OM plus *L. murinus* CR147 (OM + CR147) was analyzed by 16S rRNA gene sequencing (*n* = 7–8 for each group). Data are shown as mean ± s.e.m. Differences were assessed by Mann-Whitney *U* test (two-tailed). ***P < 0.001. (TIFF 84 kb)

## Abbreviations

ANI: Average nucleotide identity
BCS: Bacterial culture supernatant
COG: Clusters of orthologous groups
CR: Calorie restriction
ERIC-PCR: Enterobacterial repetitive intergenic consensus polymerase chain reaction
IL-8: Interleukin 8
LBP: Lipopolysaccharide (LPS)-binding protein
MANOVA: Multivariate analysis of variance
NGM: Nematode growth medium
OTU: Operational taxonomic unit
PCoA: Principal coordinate analysis
PCR-DGGE: Polymerase chain reaction/denaturing gradient gel electrophoresis
PerMANOVA: Permutational multivariate analysis of variance
QIIME: Quantitative insights into microbial ecology
RDP: Ribosomal Database Project
TNF-α: Tumour necrosis factor alpha
