# Supplementary material for: Predominant gut Lactobacillus murinus strain mediates anti-inflammaging effects in calorie-restricted mice
Source: Microbiome. 2018 Mar 21;6:54. doi: 10.1186/s40168-018-0440-5 (PMC5863386; doi:10.1186/s40168-018-0440-5)
Supplement: Supplementary file 1 — Supplementary results about summary of sequencing on the V3-V4 region of 16S rRNA gene. (DOCX 13 kb) [file 40168_2018_440_MOESM1_ESM.docx]

**Supplementary results**

**Summary of sequencing on the V3-V4 region of 16s rRNA gene**

On average, 16939 ± 5365 (mean ± standard deviation) high-quality reads were obtained for each sample. A total of 741 species-level operational taxonomic units (OTUs) were obtained at a 97% cutoff of sequence similarity using the UPARSE algorithm. Three samples were excluded for later analysis because their high-quality reads were less than 8000.
