# Supplementary figures and images for: Predominant gut Lactobacillus murinus strain mediates anti-inflammaging effects in calorie-restricted mice
Source: Microbiome. 2018 Mar 21;6:54. doi: 10.1186/s40168-018-0440-5 (PMC5863386; doi:10.1186/s40168-018-0440-5)

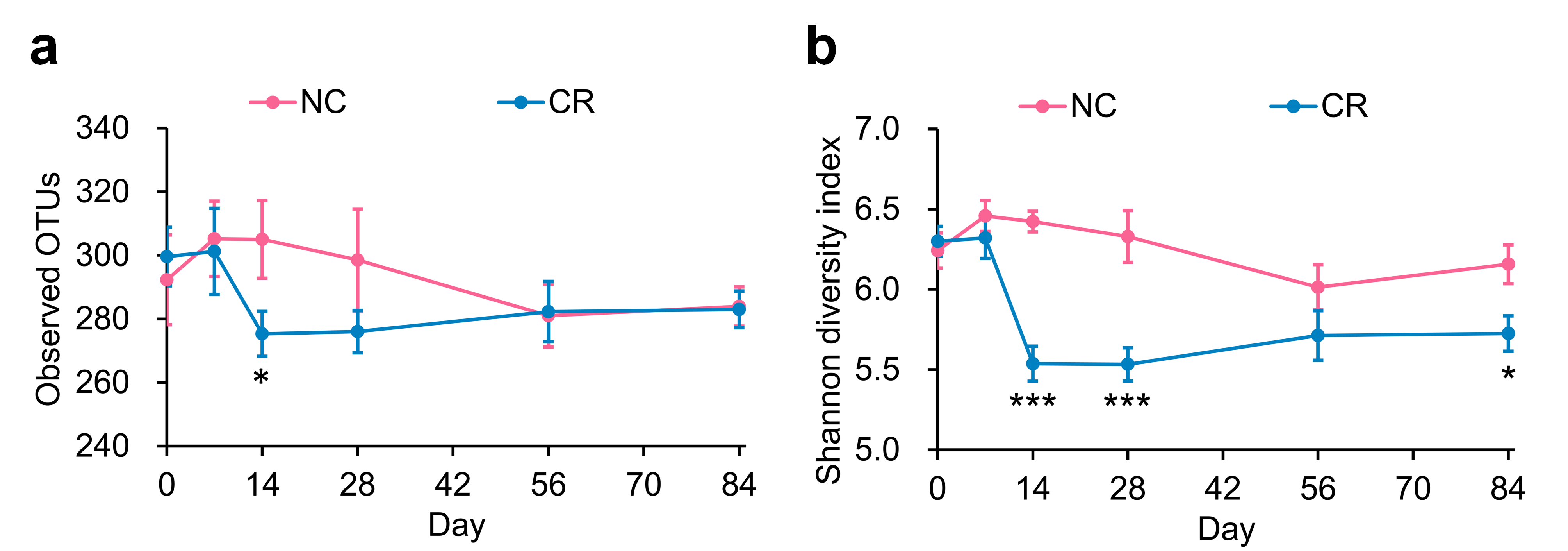

Supplement: Supplementary file 2 — Alpha diversity of the gut microbiota during short-term calorie restriction. a Observed OTUs and b Shannon diversity index at sampling level of 8000. Data are shown as mean ± s.e.m. Mann-Whitney U test (two-tailed) was used to analyse variation between the NC and CR groups at the same time point. *P < 0.05 and ***P < 0.001 vs the NC group. Sample sizes are the same as in Fig. 2. (TIFF 295 kb) [file 40168_2018_440_MOESM2_ESM.tif]

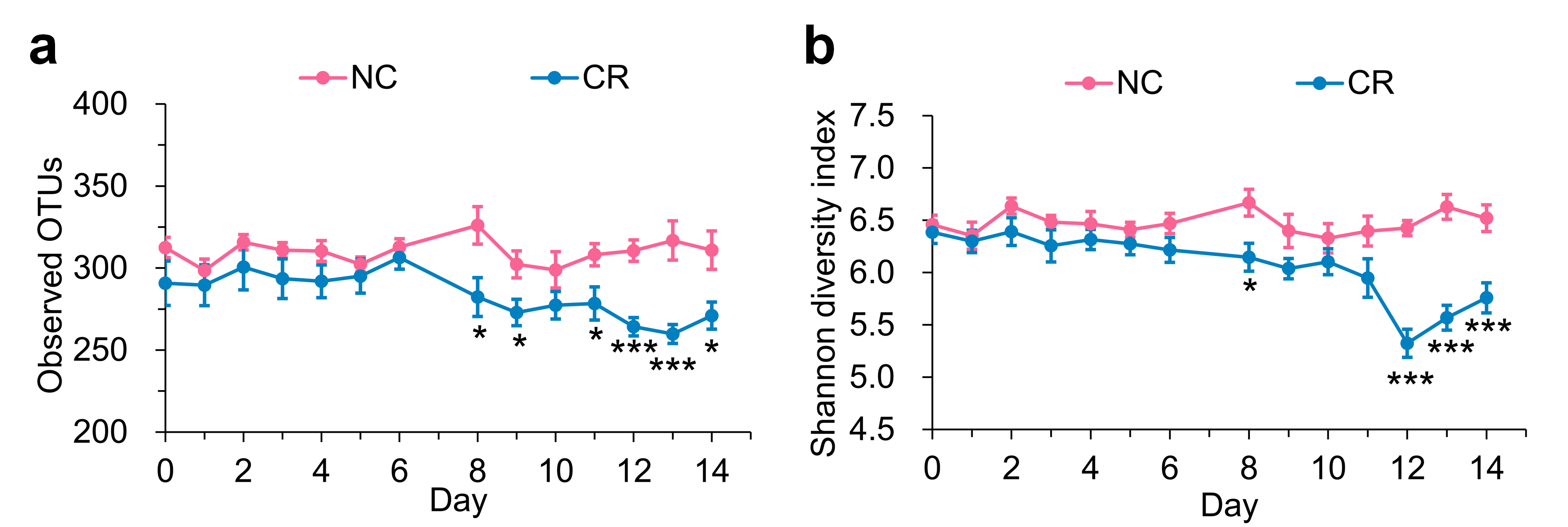

Supplement: Supplementary file 4 — Alpha diversity of the gut microbiota in the first 14 days of calorie restriction. a Observed OTUs and b Shannon diversity index at sampling level of 8000. Data are shown as mean ± s.e.m. Mann-Whitney U test (two-tailed) was used to analyse the variation between the NC and CR groups at the same time point. *P < 0.05, **P < 0.01 and ***P < 0.001 vs the NC group. Sample sizes are the same as in Fig. 3. (TIFF 317 kb) [file 40168_2018_440_MOESM4_ESM.tif]

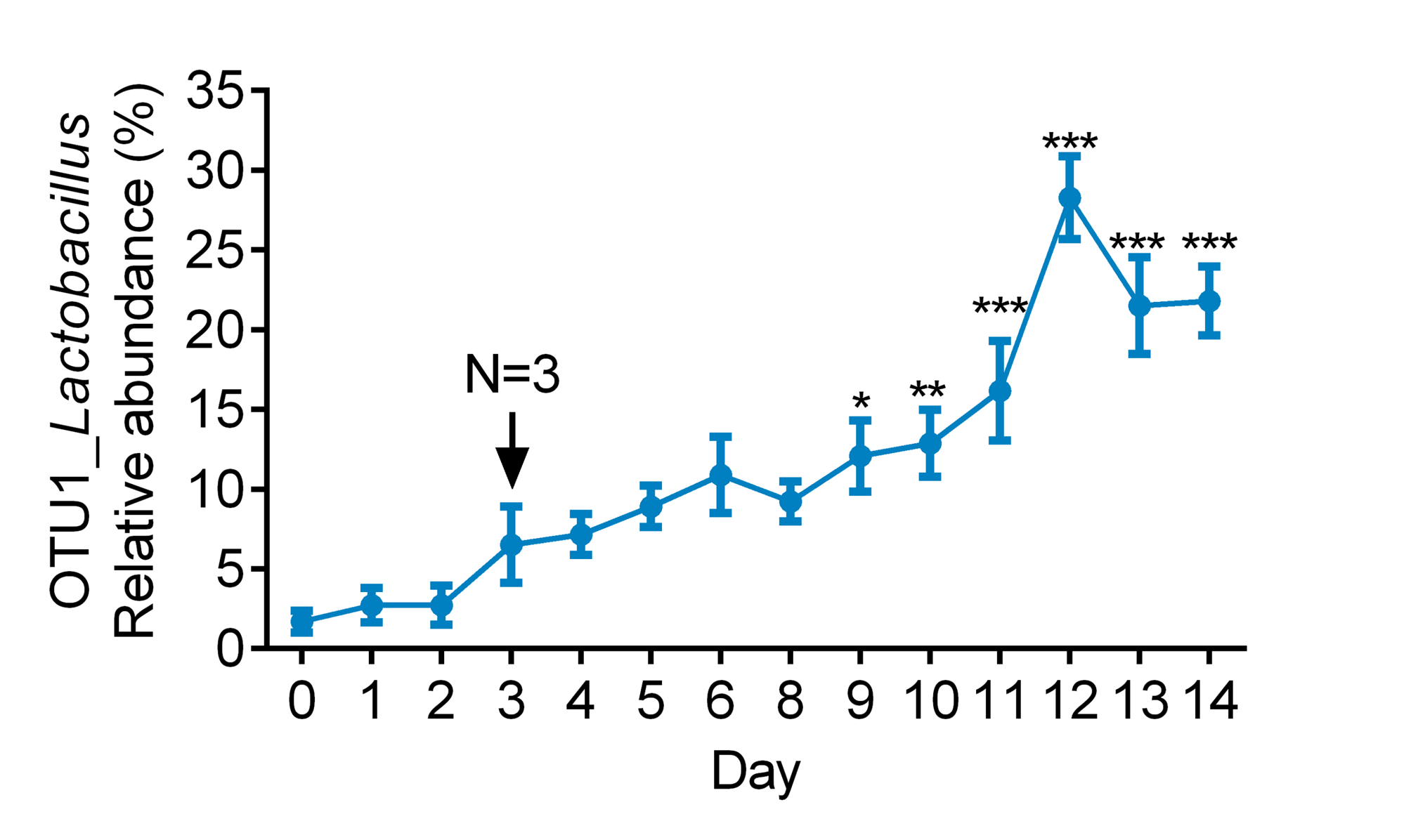

Supplement: Supplementary file 6 — Dynamics of OTU1 in CR group during the first 14 days of calorie restriction. Data are shown as mean ± s.e.m. N was the day when OTU1 began to increase, as determined by its absolute value of logarithmic (base 2) fold change in relative abundance greater than 1 (|log2-fold change| > 1), and became the most abundant phylotype in CR mice. Kruskal-Wallis test followed by Dunn’s multiple comparison test was used to analyse the variation relative to day 0. *P < 0.05, **P < 0.01 and ***P < 0.001 vs day 0. (TIFF 143 kb) [file 40168_2018_440_MOESM6_ESM.tif]

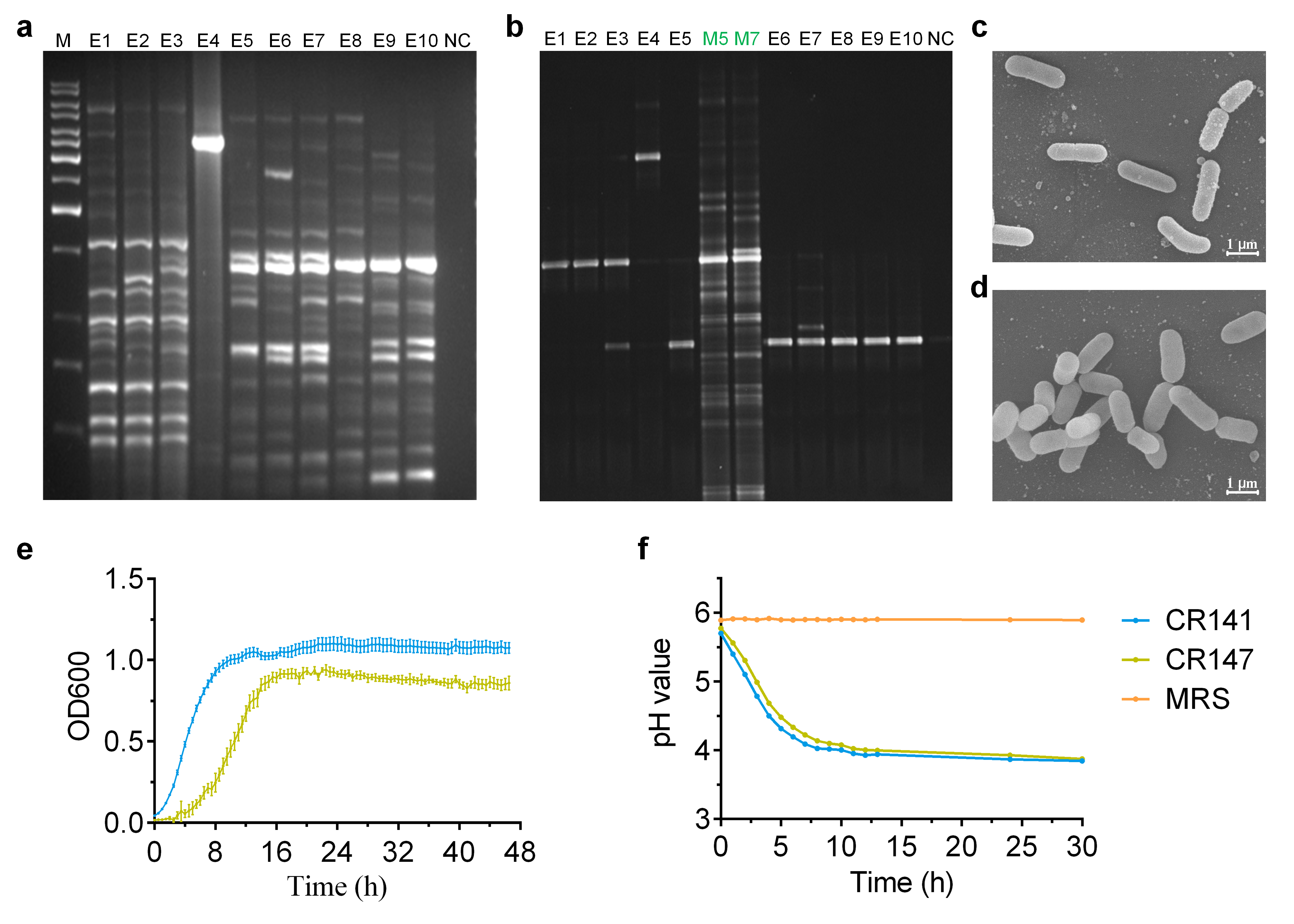

Supplement: Supplementary file 7 — Sequence-guided isolation of the predominant Lactobacillus spp. in CR mice gut. a 217 Lactobacillus isolates were classified into ten strains (E1-E10) based on ERIC-PCR profiles. M, DNA ladder. b Strains CR141 (E1) and CR147 (E2) were identified as representatives of the most abundant Lactobacillus spp. based on their co-migration pattern with the most dominant band in the fecal DNA fingerprint of CR mice in the DGGE profiles. M5 and M7, fecal DNA samples of mice subjected to CR for 2 weeks. NC, negative control. c/d Electron micrograph of strain CR141 (c) and CR147 (d). e The growth curves of CR141 and CR147 in the MRS medium. f Changes of the pH value during the growth of CR141 and CR147 in the MRS medium. (TIFF 4862 kb) [file 40168_2018_440_MOESM7_ESM.tif]

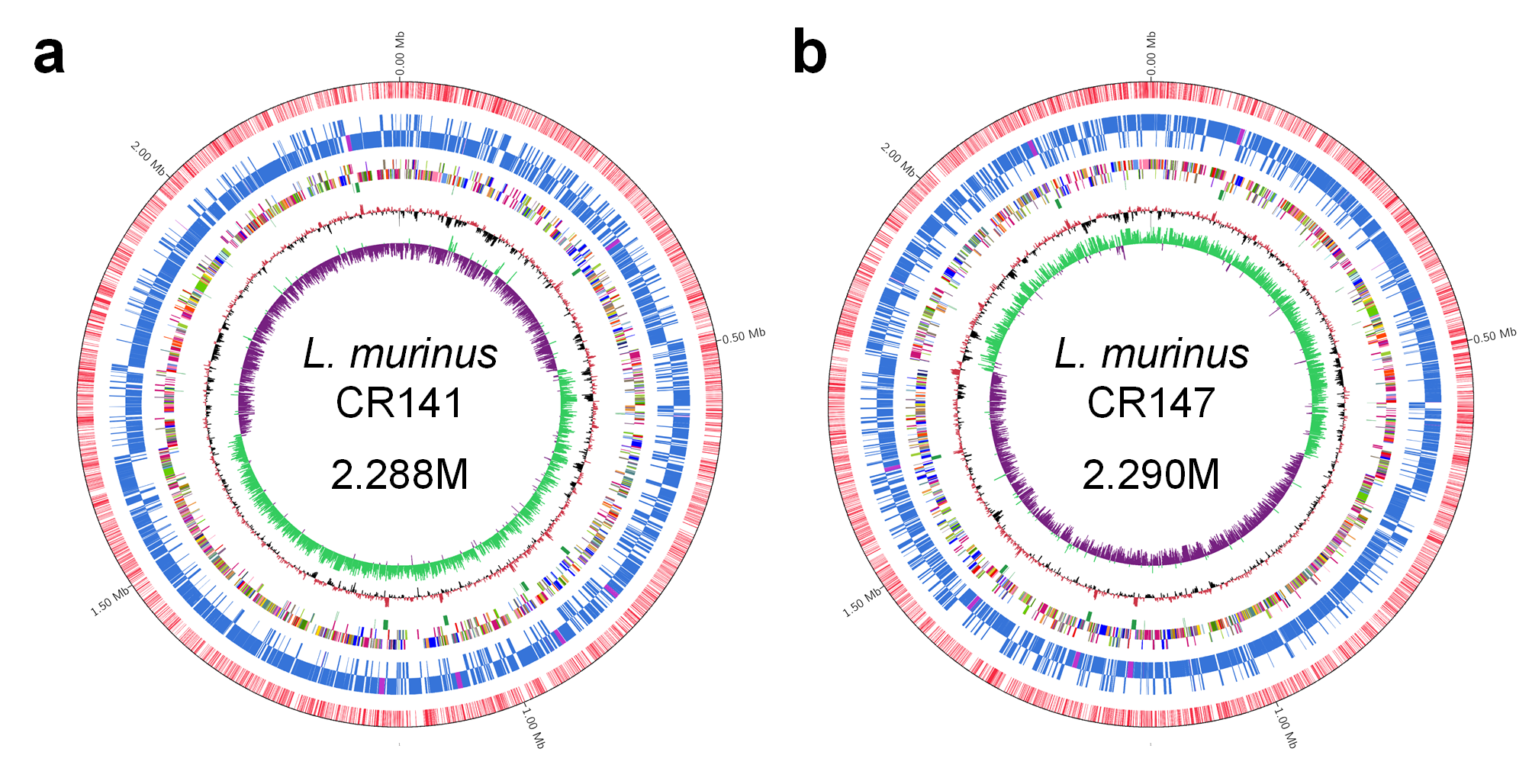

Supplement: Supplementary file 10 — Genome atlas of the two strains of Lactobacillus murinus. a L. murinus CR141. b L. murinus CR147. From inner to outer: GC skew (G − C)/(G + C), mean centered GC content (red-above mean, blue-below mean), tRNAs/rRNAs, CDS (reverse and forward strand), m4C and m6A sites in CDS/rRNA/tRNA (reverse and forward strand), m4C and m6A sites in inter-gene regions. (TIFF 1217 kb) [file 40168_2018_440_MOESM10_ESM.tif]

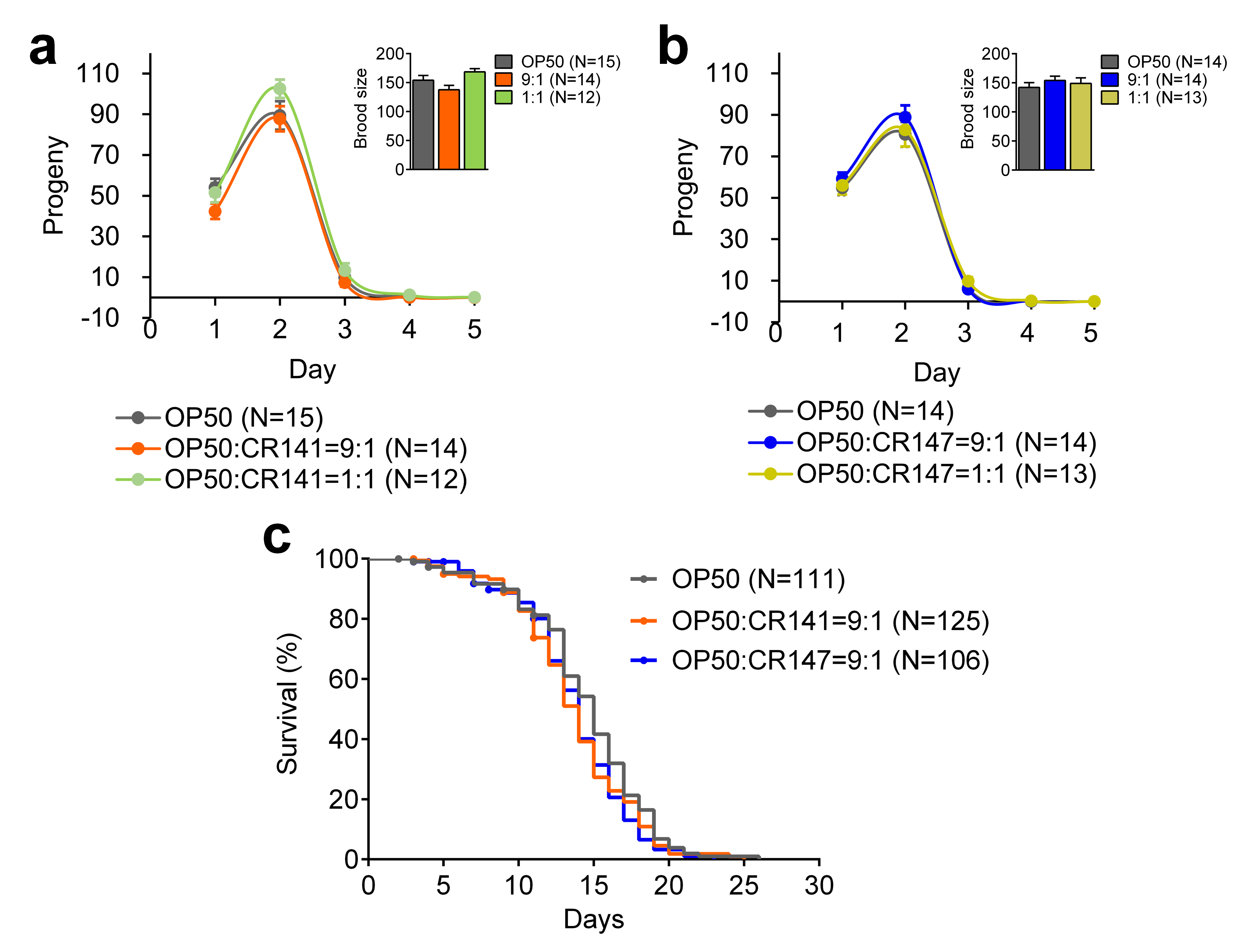

Supplement: Supplementary file 13 — Effects of L. murinus on the egg-laying schedules, brood size and lifespan of C. elegans. Worms were synchronized L4-stage larvae at day 0. The egg-laying schedules and brood sizes of worms fed 9:1 or 1:1 mixture of E. coli OP50 and a L. murinus CR141 or b L. murinus CR147. Data are shown as mean ± s.e.m. c Survival curves of C. elegans fed a 9:1 mixture of E. coli OP50 and L. murinus compared with the lifespan of the worms fed OP50 alone. Each mNGM plate contained 10 mg of bacteria (wet weight). Differences were assessed by unpaired t test (two-tailed) (a, b) or log-rank test (c). N indicates the number of worms per group. (TIFF 646 kb) [file 40168_2018_440_MOESM13_ESM.tif]

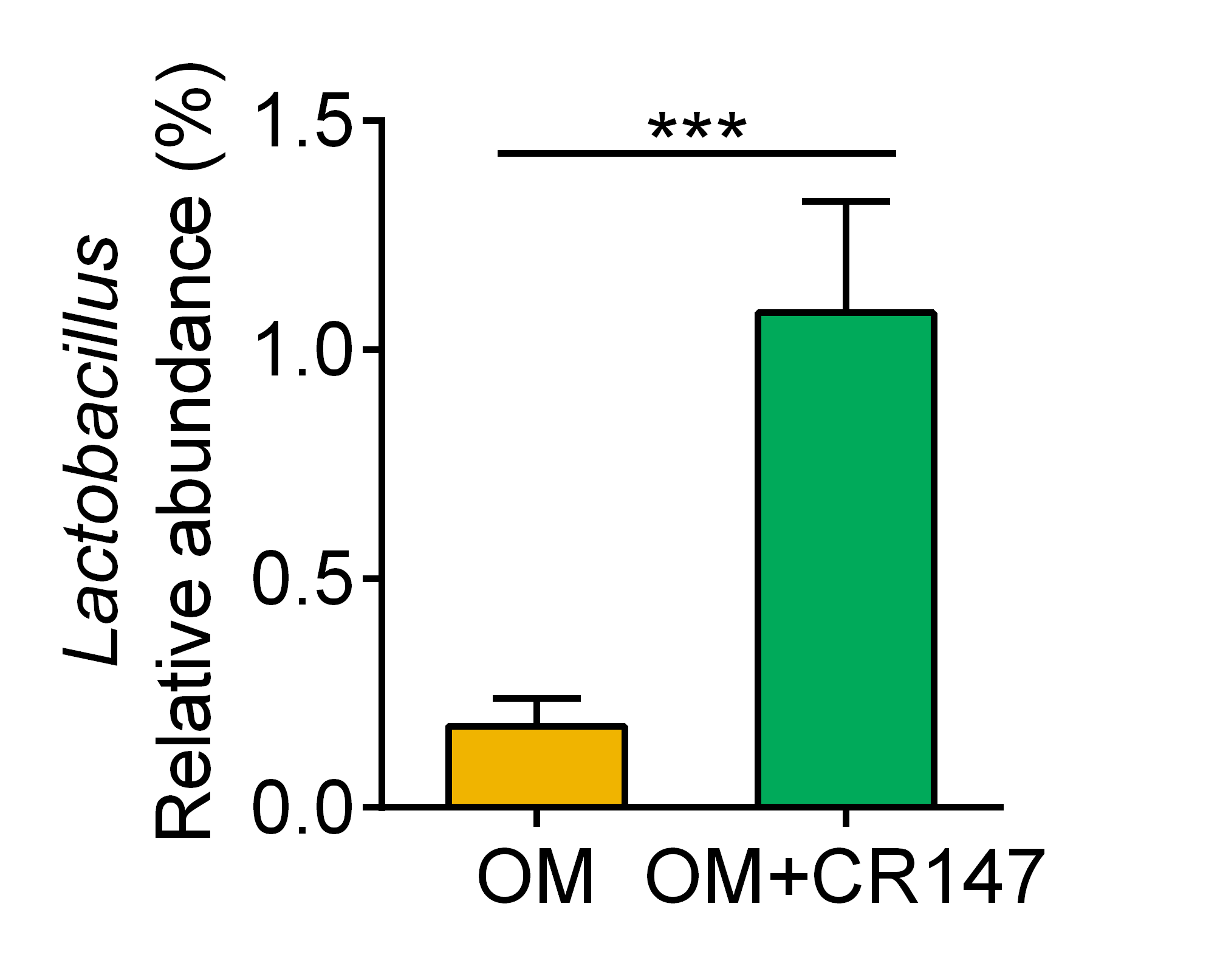

Supplement: Supplementary file 14 — L. murinus CR147 supplementation increases the abundance of Lactobacillus in old microbiota-colonized gnotobiotic mice gut. After 14 days of the inoculation, the abundance of Lactobacillus in fecal microbiota of mice colonized with old microbiota (OM group) or OM plus L. murinus CR147 (OM + CR147) was analyzed by 16S rRNA gene sequencing (n = 7–8 for each group). Data are shown as mean ± s.e.m. Differences were assessed by Mann-Whitney U test (two-tailed). ***P < 0.001. (TIFF 84 kb) [file 40168_2018_440_MOESM14_ESM.tif]
